# Antisense Morpholino Oligonucleotides Reduce Neurofilament Synthesis and Inhibit Axon Regeneration in Lamprey Reticulospinal Neurons

**DOI:** 10.1371/journal.pone.0137670

**Published:** 2015-09-14

**Authors:** Guixin Zhang, Li-qing Jin, Jianli Hu, William Rodemer, Michael E. Selzer

**Affiliations:** 1 Shriners Hospital Pediatric Research Center (Center for Neural Repair and Rehabilitation), 3500 North Broad Street, Philadelphia, United States of America; 2 Department of Neurology, Temple University School of Medicine, 3500 North Broad Street, Philadelphia, United States of America; University of Szeged, HUNGARY

## Abstract

The sea lamprey has been used as a model for the study of axonal regeneration after spinal cord injury. Previous studies have suggested that, unlike developing axons in mammal, the tips of regenerating axons in lamprey spinal cord are simple in shape, packed with neurofilaments (NFs), and contain very little F-actin. Thus it has been proposed that regeneration of axons in the central nervous system of mature vertebrates is not based on the canonical actin-dependent pulling mechanism of growth cones, but involves an internal protrusive force, perhaps generated by the transport or assembly of NFs in the distal axon. In order to assess this hypothesis, expression of NFs was manipulated by antisense morpholino oligonucleotides (MO). A standard, company-supplied MO was used as control. Axon retraction and regeneration were assessed at 2, 4 and 9 weeks after MOs were applied to a spinal cord transection (TX) site. Antisense MO inhibited NF180 expression compared to control MO. The effect of inhibiting NF expression on axon retraction and regeneration was studied by measuring the distance of axon tips from the TX site at 2 and 4 weeks post-TX, and counting the number of reticulospinal neurons (RNs) retrogradely labeled by fluorescently-tagged dextran injected caudal to the injury at 9 weeks post-TX. There was no statistically significant effect of MO on axon retraction at 2 weeks post-TX. However, at both 4 and 9 weeks post-TX, inhibition of NF expression inhibited axon regeneration.

## Introduction

The sea lamprey is a convenient model for the study of axon regeneration after spinal cord injury (SCI), in part because identified reticulospinal neurons (RNs) with known, heterogeneous regenerative abilities can be imaged in histological whole-mounts [1, 2]. Previous reports of recovery following spinal cord transection (TX) in lamprey included behavioral, histological and electrophysiological descriptions [3–10]. Mammalian models of SCI usually involve partial lesions, and enhanced axon growth and functional recovery could be due to collateral sprouting from uninjured spared axons. Thus it is difficult to determine whether manipulations that influence axon growth and functional recovery are influencing true axon regeneration or collateral sprouting by spared axons. By contrast, axon regeneration occurs spontaneously in the lamprey after complete SCI, and thus effects of molecular manipulations on regeneration across a TX are unambiguous. Furthermore, unlike developing axons, the tips of regenerating axons in lamprey spinal cord are simple in shape, packed with neurofilaments (NFs), and contain some microtubules but very little F-actin [11–14]. This calls into question the idea that regeneration recapitulates the mechanism of early axon development, and it has been proposed that regeneration of axons in the central nervous system (CNS) of mature vertebrates is not based on the canonical actin-dependent pulling mechanism of growth cones, but involves an internal protrusive force, perhaps generated by the transport or assembly of NFs in the distal axon [12, 13]. Regenerative ability among RNs assessed at 10 weeks post-TX was positively correlated with their ability to re-express NF mRNA, after transient down regulation during the first 4 weeks [2]. The early decrease in NF mRNA expression was not accompanied by a reduction in NF protein concentration by western blot, possibly because the volume of axon into which newly synthesized NF could be distributed was greatly reduced [2]. Moreover, the pattern of expression was not altered when regeneration was blocked mechanically. Thus the secondary upregulation of NF message is not a consequence of axon growth, but may be part of an intrinsic growth program executed only in neurons with a strong propensity for regeneration.

If synthesis of NFs is important for axon regeneration in the CNS, then inhibiting NF synthesis should inhibit regeneration. In the present study we therefore examined, the effect of NFs on axon regeneration by blocking the translation of a key NF subunit, NF180, with antisense morpholino oligonucleotides (MOs). We also evaluated: 1) Whether, in addition to regrowth from severed axons (true regeneration), there is collateral sprouting from spare axons after hemisection of lamprey spinal cord; 2) NFs protein expression and time course of expression in lampreys of different sizes (and therefore different ages) for up to 1 year after spinal cord TX. These features of the response to spinal cord injury are very important for using lamprey as a model for axonal regeneration and for using expression of NFs as a marker of axonal regeneration.

## Materials and Methods

### Ethical Statement

This study was carried out in strict accordance with the recommendations in the Guide for the Care and Use of Laboratory Animals of the National Institutes of Health. The protocol was approved by the Institutional Animal Care and Use Committees of the University of Pennsylvania (Protocol# 063600) and Temple University (Protocol# 4252). All surgery was performed under aqueous benzocaine anesthesia, and all efforts were made to minimize suffering.

### Animals and spinal cord transection

Larval sea lampreys (*Petromyzon marinus*), 10–12 cm in length (3 to 4 years old), were obtained from commercial suppliers in Michigan, USA, and maintained in freshwater tanks at 16°C until the day of use. Lampreys were anesthetized by immersion in saturated aqueous benzocaine for several minutes and then pinned to a Sylgard- 184 silicone elastomer plate (Dow Corning, Midland, Michigan) filled with ice-cold lamprey Ringer (110 mM NaCl, 2.1 mM KCl, 2.6 mM CaCl2, 1.8 mM MgCl2, and 10 mM Tris buffer; pH 7.4). The spinal cord was exposed via a dorsal incision and transected at the level of the 5th gill. Animals were covered with a lamprey Ringer-moistened paper towel and kept on ice for 1 hour to facilitate clot formation. After returning to fresh water and remaining at 4°C overnight, they recovered at room temperature for various times (up to 1 year) before their NFs were studied by immunohistochemistry (see below).

### Tissue collection and processing

At the end of an experiment, animals were deeply anesthetized in saturated aqueous benzocaine and killed by removing the brain and spinal cord for histological processing as described below.

### Testing for collateral sprouting

Although regeneration occurs in lamprey spinal cord after complete TX and the lamprey is not often used to study partial injuries, we determined whether axons of reticulospinal neurons (RNs) undergo collateral sprouting, as after mammalian spinal cord injury, and, if so, whether we could distinguish collateral sprouting from true regeneration (see below). We therefore performed a unilateral spinal cord hemisection at the level of the 5^th^ gill (n = 4). Four weeks later these spinal cords were completely transected 1 cm caudal to the original hemisection, and dextran, Alexa Fluor 488 (DAF 488, 10 kDa, 5% in 0.1 M Tris buffer, pH 7.4, Molecular Probes) was applied at the TX site to label axons (and their perikarya) that had not been injured by the hemisection. Axons labeled in this fashion include those that had been spared by the hemisection and may have undergone collateral sprouting, and others that may have regenerated after the hemisection. To distinguish between these two groups, it was necessary to label axons that had been cut by the hemisection. A Gelfoam plug soaked in dextran tetramethyl-rhodamine (DTMR, 10 kDa, 5% in 0.1 M Tris buffer, pH 7.4, Molecular Probes) was therefore inserted into the hemisection site at the time of the original surgery. Five weeks later a second dye (DAF 488) was applied to a complete TX 1 cm caudal to the hemisection, as described above. Following an additional 1-week recovery period, the brain and spinal cord rostral to the second TX were removed, the choroid plexus stripped off to uncover the brain ventricles and the cerebrotectal commissure cut along the dorsal midline. The tissue was then pinned flat to a small strip of Sylgard, fixed 3 to 4 hrs with 4% paraformaldehyde (PFA) in phosphate buffer saline (PBS) and whole-mounted in ProLong Gold antifade reagent. Collateral sprouting was assessed using conventional fluorescence (Nikon 80i) and two-photon microscopy to identify branches crossing the midline to innervate neurons that had been denervated by the original hemisection. The 2-photon microscope used a Chameleon Ti:Sapphire laser (Coherent, Santa Clara, CA) coupled to an Ultima 2-photon scanhead (Prairie Technologies, Middleton, WI).

### Whole-mount immunohistochemistry for NFs

We used whole-mount immunohistochemistry to investigate the expression of NFs protein at different recovery times after spinal cord TX. This group consisted of a total of 36 lampreys that recovered from 1 day to up to 1 year (Table 1). The brain and spinal cord were removed, pinned flat to a strip of Sylgard and fixed in 4% PFA, as described above. The specimens were washed 3 times in PBS for 30 min each, followed by immersion in 70% ethanol at 4°C overnight and storage at -20°C for future use. The samples were then washed 3 times in PBS containing 0.1% Triton X-100 (TPBS) for 30 min each and digested in 0.1% collagenase for 1 min. The whole-mounted tissues were washed again with TPBS, blocked in 10% fetal calf serum (FCS) for 30 min and incubated with a phosphorylation-independent monoclonal antibody (mAb, LCM16, 1:100) in blocking solution for 2 days at 4°C on a rotator. This antibody is specific for the lamprey subunit NF180. After the tissues were washed and blocked again, the primary antibody was detected using the Avidin-Biotin Complex (ABC) Kit as described in the company manual (Biomeda, Foster City, California), except for incubation in secondary antibody overnight at 4°C and development in metal enhanced DAB substrate (Pierce, Rockford, Illinois). Finally the tissues were dehydrated in serial dilutions of ethanol, cleared in toluene, and mounted with Permount. To study the expression of NFs at different developmental stages, larvae of 7 and 13.5 cm in length and one young adult (14 cm) were included.

**Table 1:** Numbers of animals used for each experiment.

Experiment	Ctr	1 day	2 days	3 days	5 days	7 days	2 wks	5 wks	10 wks	17 wks	6 mths	1 yr	Total
**NF180 IHC**	**2**	**2**	**2**	**2**	**2**	**2**	**2**	**5**	**5**	**5**	**5**	**2**	**36**
	**4 wks-1 dye**	**5 wks-2 dyes**	**Total**										
**Hemi-TX**	**4**	**2**	**6**										
	**2 wks**	**4 wks**	**9 wks**	**Total**									
**Ctr-MO**	**8**	**8**	**3**	**19**									
**NF180-MO**	**8**	**8**	**3**	**19**									

Times in the top row are in days (d), weeks (w) or year (yr) after spinal cord transection (TX). Ctrl, control; Tot, total; IHC, immunohistochemistry; MO, morpholino antisense oligonucleotide. In the 4-week experiment, a single dye (DAF 488, green) was applied to a complete TX 4 weeks after, and 1 cm caudal to an unlabeled hemisection (hemi-TX) to look for axon collaterals crossing the midline. In the 5-week experiment, one dye (DMTR, red) was applied to a hemisection, and then 4 weeks later, a second dye (DAF 488, green) was applied to a complete TX 1 cm caudally.

### MOs application

We used an improved method for inhibition of expression based on antisense MO [15]. MOs are nonionic DNA analogs in which MOs (1-oxa-4-azacyclohexane) joined by phosphorodiamidate linkages are substituted for riboses. Each MO subunit contains one of the four genetic bases, which determines the specificity of its binding to target mRNA. Antisense MOs can form RNA-MO hybrids, but these are not substrates for RNase H and thus are not degraded. Because hybrids formed in the coding region will be displaced by the ribosome, the most effective regions for MO targeting are in the 5’-untranslated region or the region including the start codon. To determine the effect of inhibiting NF synthesis on early axon retraction and regeneration, fluorescently conjugated MOs were synthesized (Gene Tools) based on the antisense sequence “gtggTACTCGATGTGTCTCAGCTCG” (-4 to +21) spanning the translation-initial codon of lamprey NF subunit NF180 (GenBank Accession No. U19361) and the 5’ non-translation region. The NF180-MO was fluorescein-conjugated, and a rhodamine-conjugated standard control MO of the same size (5’-CCTCTTACCTCAGTTACAATTTATA-3’) that was provided by the company was used as a reference. Thirty-eight larval lampreys were anesthetized in benzocaine and their spinal cords transected at the level of the 5th gill. MOs dissolved (2%) in dH_2_O were delivered to RNs by retrograde transport from a soaked Gelfoam pledget applied acutely to the TX site. Animals recovered on ice for 2 hours, then in fresh water tanks at 21°C for 2 weeks (time of maximal axon retraction), 4 weeks (when regenerating axons reach the injury site) and 9 weeks (after regenerating axons have passed the injury site). The animals used in MO experiments are summarized in Table 1.

### Fluorescence imaging of RNs in brain and their axons in the spinal cord

To evaluate the effect of MOs on NF180 protein synthesis, two lampreys from each group of 2-week and 4-week survival were re-anesthetized, and their brain and spinal cord removed for western blotting (see below). The remaining animals were used in immunohistochemistry studies and to determine the role of NF180 in axonal regeneration. The brain and spinal cord of 2- and 4-week survivors (6 lampreys in each group) were removed, pinned flat to a strip of Sylgard and fixed in 4% PFA in PBS, as above. To visualize RNs retrogradely labeled with fluorescently-tagged MOs, the brain was viewed and photographed with a fluorescence microscope (80i, Nikon). The spinal cord also was photographed, in order to evaluate the effect of MOs on axon retraction and on the early phase of regeneration, when back-labeled large reticulospinal axons are easily identified in the proximal (rostral) stump. The distance between the axon tips, which remained brightly labeled by the fluorescently-tagged MOs, and the TX site was measured. Only axons with clearly identified tips were analyzed. All samples were then processed for immunohistochemistry for NF180 to confirm that MOs had blocked the synthesis of NF180. To test the long-term effect of MOs on axon regeneration beyond the lesion site, animals that had received control- or NF180-MOs (n = 3, each group) were allowed to recover for 9 weeks after application of the MOs. A second TX was then made 5 mm caudal to the original TX, and a second label, 5% DAF 488 (for control-MO) or DTMR (for NF180-MO), was applied to the TX site to label neurons whose axons had regenerated at least to this level. One week later the brain and spinal cord rostral to the second TX were removed, pinned flat to a strip of Sylgard, fixed in 4% PFA and whole-mounted in ProLong Gold antifade reagent. The brains were then photographed with a fluorescence microscope (80i, Nikon) and analyzed. Double-labeled RNs (*i*.*e*., those that were labeled at the original TX with fluorescently-tagged MOs, and also labeled from the second TX and therefore had regenerated) from each group were counted, and the difference between the two groups compared and analyzed by ANOVA and post-hoc two-tailed t-test.

### Western blotting

Lampreys that had survived either 2 or 4 weeks (two animals at each time) after the application of MOs described above were re-anesthetized and their brain and spinal cord rostral to the TX removed. The tissues were snap frozen by liquid nitrogen and homogenized by sonication in cold HEPES buffer (1 mg wet wt./10 μl), followed by centrifugation at 500×*g* for 10 min. After one tenth of the supernatant, which contained unassembled NF subunits, had been collected, the remainder was spun at 18,000×*g* for 10 min. The pellet, which contained assembled NFs, was re-suspended in HEPES. Protein (50 μg wet wt./lane in supernatant and 100 μg wet wt./lane in pellet) was resolved in a 10% SDS acrylamide gel and transferred to nitrocellulose membranes using a Bio-Rad transblot apparatus. The membranes were then preincubated in 5% nonfat dry milk in Tris-buffered saline (TBS) and blotted with a mixture of LCM-39 (for NF180) and LCM-40 (nIF50), another neuronal intermediate filament subunit. After overnight incubation with mAbs at 4°C, blots were washed with 0.1 M TBS containing 0.1% Tween-20, and then incubated with horseradish peroxidase-conjugated anti-mouse IgG secondary antibody (Santa Cruz Biotechnology, Santa Cruz, CA) for 1 hour. After washing with 0.1 M TBS for 30 minutes, immunoreactive proteins were visualized with the enhanced chemiluminescence (ECL) kit.

## Results

### Recovery of lamprey from SCI is accompanied by axon regeneration, not by collateral sprouting

**Fig 1:**
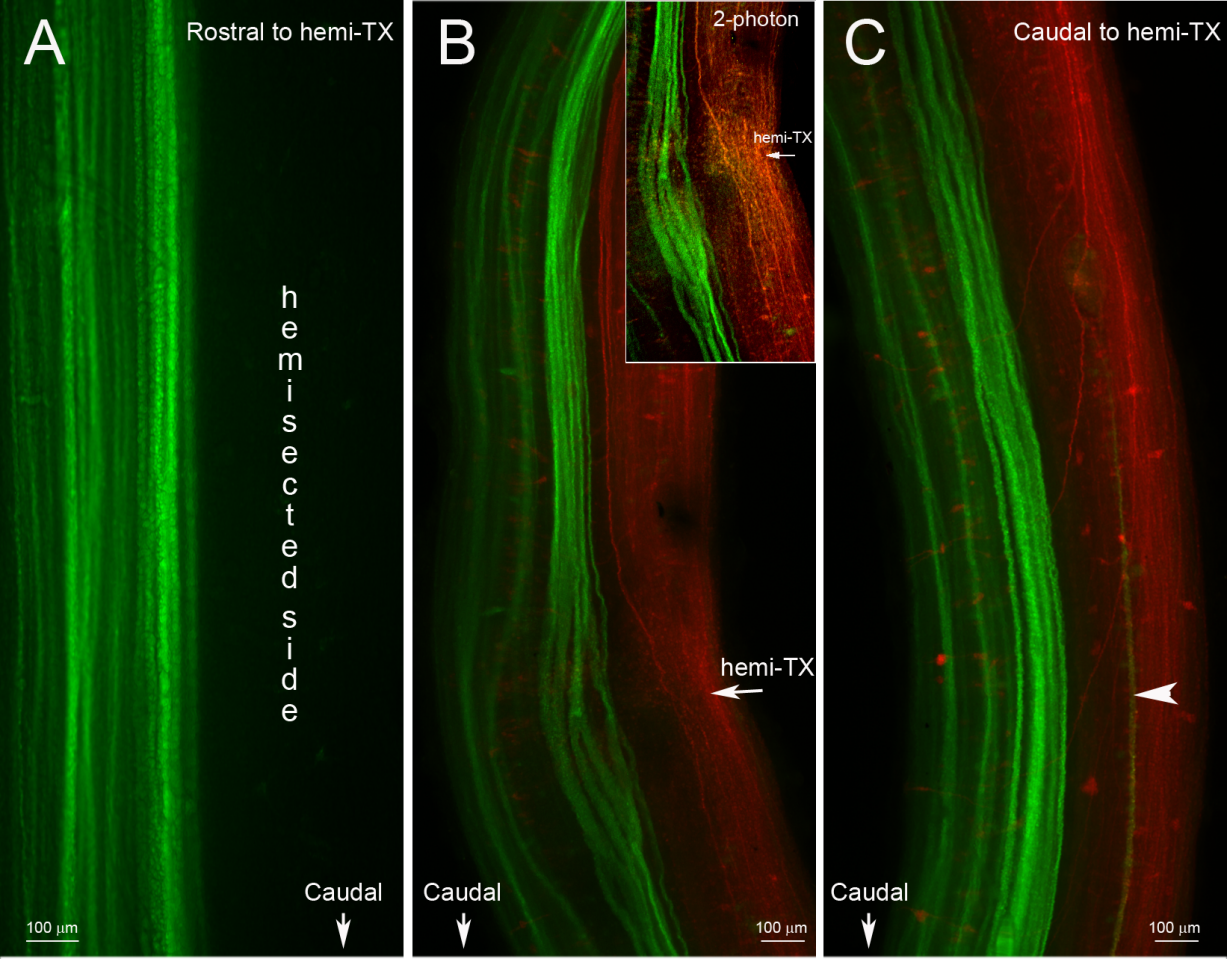
No collateral sprouting from spared spinal cord axons at 4 or 5 weeks after a hemi-TX. **A,** unilateral labeling of previously uninjured axons in a wholemounted spinal cord rostral to a right-sided hemi-TX. Four weeks after the hemi-TX, a green dye (DAF 488) was applied to a complete TX 1 cm caudal to the hemi-TX. Axons severed by the hemi-TX were not labeled, while the originally non-injured axons were labeled in green. No decussating sprouts are seen. **B** and **C,** wholemounted spinal cord of an animal rostral (**B**) and caudal (**C**) to the hemi-TX site at the level of the 5^th^ gill (arrow in **B**). DTMR (red) was applied to the hemi-TX, labeling the severed axons and their cell bodies. Five weeks later, a complete TX was made 1 cm caudal to the hemi-TX and DAF 488 (green) was applied, labeling the previously spared axons. The inset in **B** is a 2-photon photomicrograph of the hemi-TX region, showing axons regenerating across the hemi-TX. **B** and **C** show no evidence of collateral sprouting by the green-labeled (previously spared) axons. A single longitudinal green fiber in **C** (arrowhead) is the decussated rostrally-projecting axon of a giant interneuron whose perikaryon is located caudal to the depicted level.

**Fig 2:**
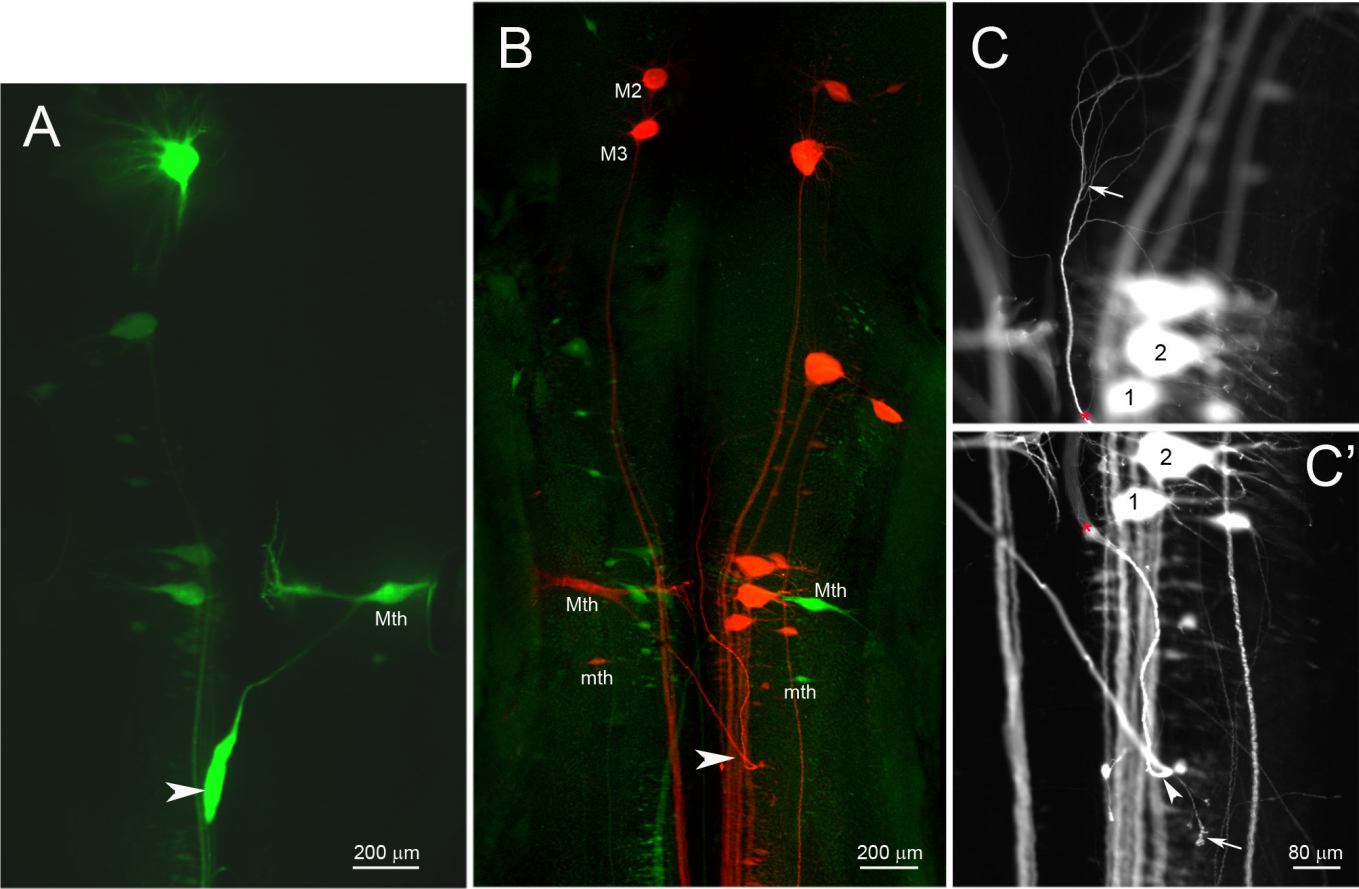
Retrograde labeling of RNs in hemisected animals. Animals were subjected to spinal cord unilateral hemi-TX followed by complete TX caudal to the hemi-TX, as in Fig 1. **A,** DAF 488-labeled RNs, whose axons were not originally injured by a right-sided hemi-TX. The right Mauthner (Mth) neuron is labeled because its axon decussates in the brainstem and projects to the contralateral spinal cord. In this case, its axon (arrowhead) had rapidly retracted to just below its decussation in the floor of the 4th ventricle. **B**, dual retrograde labeling of RNs. Those axotomized by a right-sided hemi-TX were back-labeled with DTMR (red), while those that were spared are labeled by DAF 488 (green) applied to a complete TX 5 weeks later. Mth and auxiliary Mauthner (mth) neurons have decussating axons and are labeled contralateral to other large (Müller) neurons. (The left M3 and M2 neurons were unintentionally labeled red because their axons are located near the spinal cord midline and were injured by the hemi-TX.) A Mth axon severed by the original hemi-TX (labeled red) had retracted to the 4^th^ ventricle and looped rostrally (arrowhead). This is shown at higher magnification in **C** and **C’**, in which the rostrally oriented axon and its terminal branches are followed at two focal planes. Numbers identify the same neurons, while the * indicates the same point on the axon. The point of axon looping is indicated by the arrowhead in **C’**. Arrows in **C** and **C’** point to terminal branches and sprouts emanating from the looping point, respectively.

In the unilateral hemisection experiment, uninjured axons were labeled by applying a green fluorescent dye (DAF 488) to the site of a complete TX made 4 weeks after and 1 cm caudal to the initial lesion. The descending axons belonging to identified RNs are normally unbranched and range from 20 to 40 μm in diameter. Most of them, including the giant Müller axons project ipsilaterally. Some, including the Mauthner axon decussate in the brainstem and project contralaterally, but in control animals, all the large reticulospinal axons continue unbranched the length of the spinal cord. Thus they are easily visible in spinal cord whole-mounts. If they exhibited collateral sprouting, we would expect to see green-labeled axons sending a branch contralaterally to innervate the hemisected side, where many neurons had lost synaptic inputs. No such branches were observed. Thus we found no evidence for collateral sprouting either rostral (Fig 1A) or caudal (Fig 1C) to the hemisection site, even though many axons had regenerated through the original hemisection (Fig 1B). Some transected axons retracted for long distances. Generally they belonged to “bad regenerating” neurons, such as the Mauthner cell (Mth), which in Fig 2A has retracted all the way to the level of the 4th ventricle (arrowhead), M3 and B1 (not shown). The double labeling experiment illustrated by Fig 1B and 1C demonstrates that axons regenerated through the region of the hemisection (inset in Fig 1B), but decussating collateral sprouts were not observed. Some axons turned rostralward from the hemisection, and some axons gave rise to more than one branch either rostral to or caudal to the hemisection. As reported previously, some of the RNs of lamprey undergo a very delayed form of neuronal death [16], but at 5 weeks after injury, most of them were still present, although they were swollen (red) compared to those whose axons were injured freshly (green). In Fig 2B and 2C, the left-sided Mth neuron exemplifies three interesting response to chronic axotomy: 1) Its axon, injured 6 weeks previously, retracted back to the 4^th^ ventricle; 2) this axon then looped to grow rostralward (arrowheads in Fig 2B and 2C’); and 3) the axon branched (arrow in Fig 2C). Small, correctly oriented, sprouts appeared at the turning site (arrow in Fig 2C’).

### Developmental increase in expression of NF180 proteins in lamprey brain

**Fig 3:**
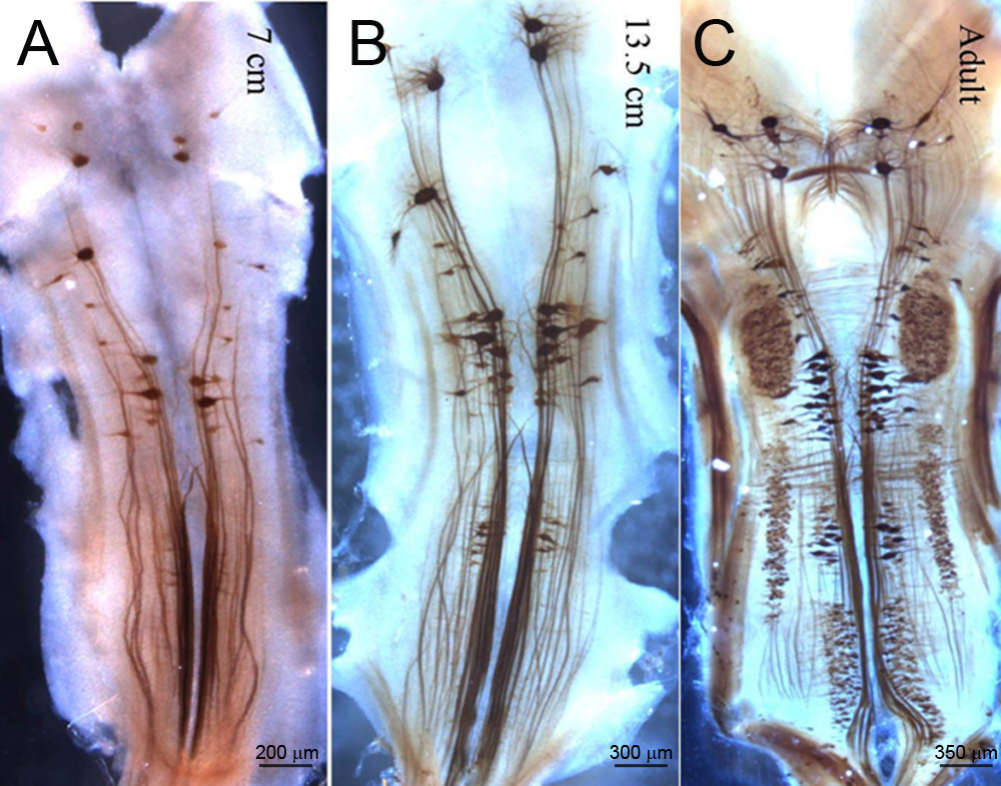
Developmental increase in NF180 protein expression in lamprey brain. Immunohistochemistry for NF180 in brain whole-mounts show a progressive increase in the number of labeled RNs with age, from approximately 3 years (7.5 cm) to 4 years (13.5 cm) to postmetamorphic adult.

We previously reported developmental increases in expression of NF mRNA in RNs of lamprey by *in situ* hybridization [17]. Therefore, in order to compare anti-NF MO-treated neurons with controls, it was necessary to examine the effect of age on expression of NF180 protein in lampreys, so that we could match MO-treated and control animals appropriately by age. Consistent with the observed mRNA expression changes, we observed an increase in NF180 protein expression in RNs during development. The intensity of labeling for NF180 protein in animals < 10 cm (2–3 years old) varied among neurons and ranged from no label to very strong (Fig 3A). In lampreys 13 cm in length (4–5 years old) most of the 18 pairs of identified RNs revealed staining for NF180 (Fig 3B) that was more intense and less heterogeneous than in younger larvae. Both the intensity of NF180 protein expression and the number of brain neurons that expressed it increased further after metamorphosis to the young adult stage (Fig 3C).

### Changes in NF180 protein expression in axotomized central neurons

**Fig 4:**
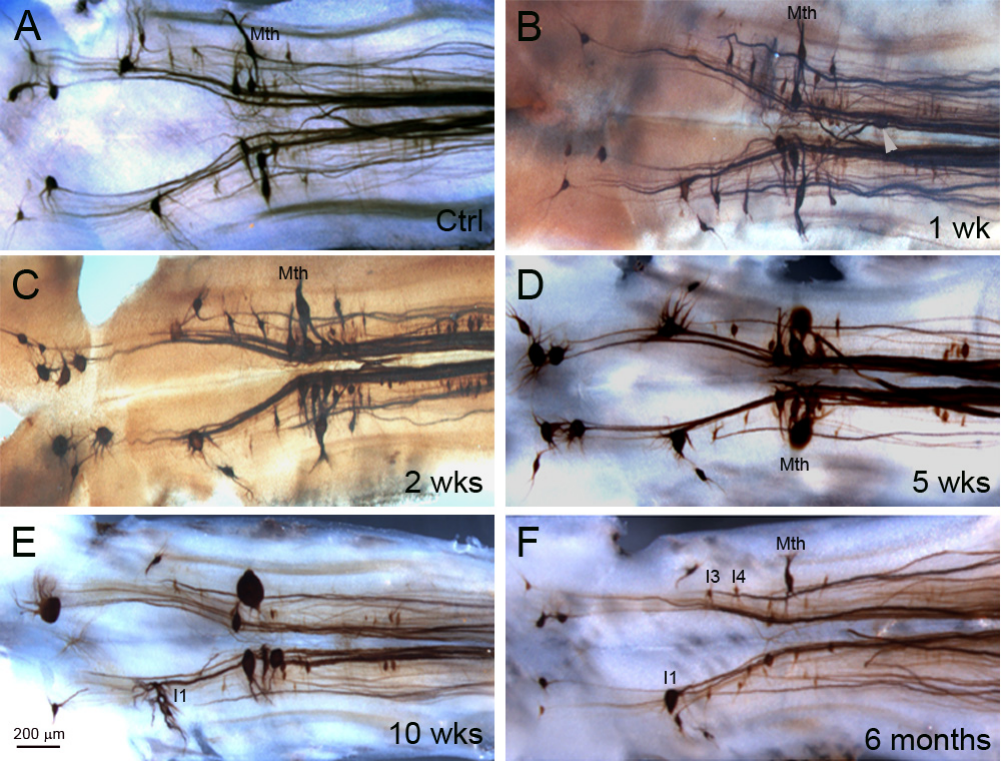
Post-TX changes in NF180-labeled neurons. In animals 11–12 cm long, the majority of identified RNs express NF180. The intensity of NF staining and the shapes of neurons did not change dramatically compared to control brain during the first 2 weeks post-TX (**A-C**). Arrowhead in B points to a Mth axon stump, showing extreme retraction after injury. By 5 weeks post-TX, perikaryal swelling and dendritic shortening and hypertrophy, were seen primarily in bad-regenerating neurons (**D-E)**. This lasted at least 17 weeks. At 6 months post-TX (**F**), most of the identified neurons that had shown dramatic swelling previously were no longer seen.

Previously, we demonstrated that the large identified RNs strongly express NF180 mRNA and protein, but whereas good-regenerating neurons recovered NF180 mRNA expression after an initial downregulation, bad-regenerating neurons failed to do so for at least 10 weeks post-TX [2]. In that study, the expression of NF180 protein post-TX was investigated by western blot, but not in by immunohistochemistry of individual RNs. Because western blots indicate total tissue protein levels, they may not distinguish changes in individual RNs. The effects of axotomy on neuronal protein levels could be underestimated if a significant amount of NF is found in cells that were not axotomized by the TX (*e*.*g*., cranial motor neurons). Moreover, at the time we did not realize that the bad regenerators often undergo a very delayed (16 weeks) form of apoptosis post-TX [16]. Therefore, in the present study, changes in NF180 protein were evaluated immunohistochemically from 1 day up to 1 year after spinal cord TX. A total of 36 animals were studied at different recovery times (Table 1). Fig 4 illustrates the changes in expression of NF180 protein after spinal cord TX. The earliest visible response to axotomy was formation of beadlike varicosities along the axons at 2 days post-TX, followed by the appearance of multiple vesicle-like structures in RNs (not shown). These changes often appeared in neurons previously determined to have poor regenerating ability (*e*.*g*., M3, M2, I1 Mth [2]). The morphology and NF180 protein contents of spinal-projecting neurons did not change noticeably during the first 2 weeks (Fig 4B and 4C). By 5 weeks, large RNs demonstrated swollen perikarya, retracted dendrites and reduced NF180 staining (Fig 4D and 4E). These changes persisted for at least 17 weeks. After 6 months, many previously swollen neurons disappeared. Identified RNs with high regeneration probabilities (*e*.*g*., I3 and I4) were usually NF180-positive at long survival times although NF180 protein levels appeared to be reduced (Fig 4F).

**Fig 5:**
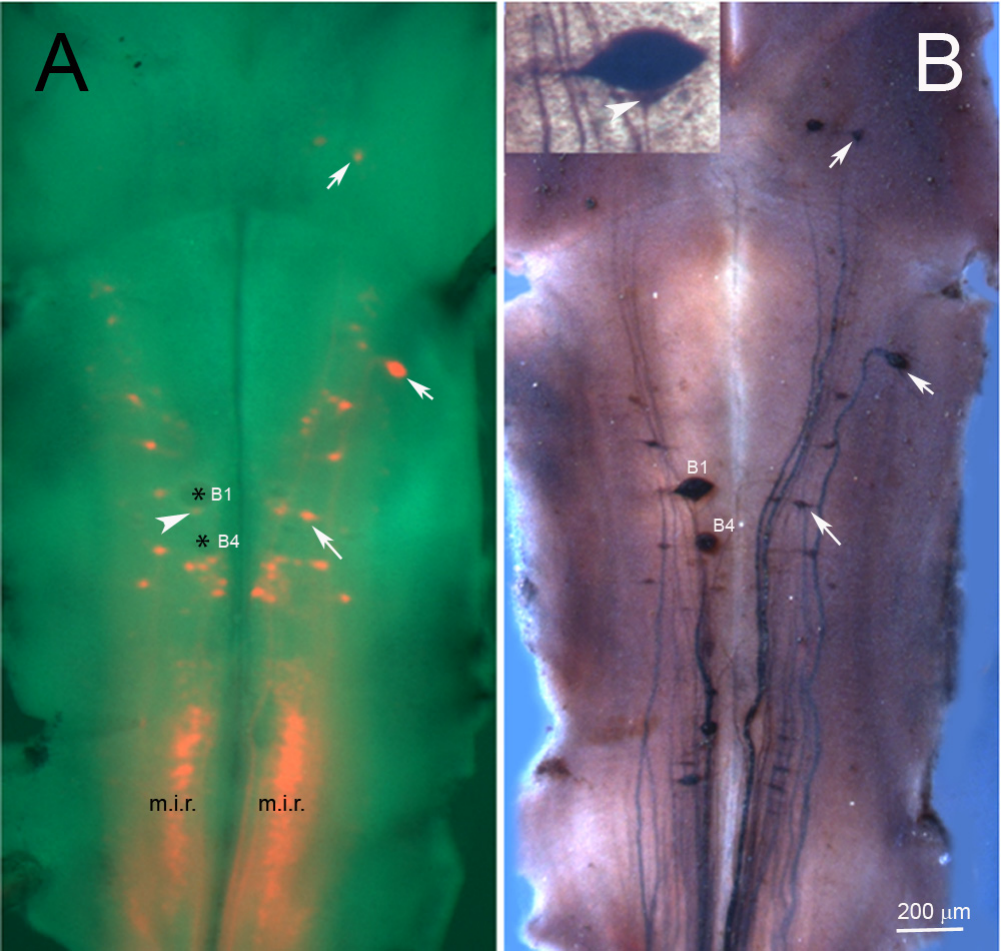
Surviving neurons continue to express NF180 protein after axotomy. In an animal 10 months post-TX at the level of the 5^th^ gill, DTMR was applied to a second complete TX at the level of the 3rd gill (approximately 5 mm below the obex of brain and 2 mm rostral to the original TX site), in order to label neurons projecting to the spinal cord, regardless of whether their axons had regenerated. After 5 days to allow for retrograde labeling, the brain was removed, pinned flat, and photographed live before fixation. **A,** fluorescently-labeled RNs superimposed on a brightfield image of the living brain. **B,** After fixation, the same brain was stained for NF180. DTMR labeling correlated closely with NF180 immunostaining, but there were exceptions: 1) Two swollen neurons (* in **A**; probably the left B1 and B4) were labeled by anti-NF180 but were not backfilled by DTMR, indicating that they had survived but their axons had retracted long distances and had not regenerated back to the level of the 3^rd^ gill. 2) Some neurons were labeled by DTMR but not by NF180. Prominent among these were neurons in the medial inferior reticulospinal group (m.i.r.), reflecting that not all spinal-projecting neurons express NF180 in animals of this age [17]. The faint stain just caudal to B1 (white arrowhead in **A)** is a small neuron, not collapsed B1 cytoplasm, as shown in the inset of **B**. The white arrows in **A** and **B** point to neurons that are double-labeled because they have axons that regenerated and also express NF180.

Because assembled NFs are comparatively stable, NF protein levels might persist, even in bad-regenerating neurons, long after mRNA expression is reduced. This was evaluated in an animal transected at the level of the 5^th^ gill as usual. Ten months later, DTMR was applied to a second TX at the level of the 3^rd^ gill, *i*.*e*., 2 mm rostral to the original TX. In this way, neurons that had survived the first transection would be labeled regardless of whether their axons had regenerated past the initial TX. We then processed the brain for NF180 immunohistochemistry. As seen in Fig 5, most of the NF+ neurons were spinal-projecting. However, not all spinal-projecting neurons were NF180+. In particular, many neurons of the medial inferior reticulospinal nucleus showed little NF180 staining. A few strongly NF180+ neurons were not retrogradely labeled from the level of the 3^rd^ gill. In Fig 5A, the B1 and B4 RNs are seen as large empty ghosts (asterisks). Yet they are intensely labeled for NF180 (Fig 5B). It is likely that their axons had retracted and had not grown back to the level of the 3^rd^ gill. In summary, most large RNs continued to express NF180 protein if they survived TX, regardless of whether their axon regenerated.

### Blocking NF180 synthesis with antisense MOs inhibits regeneration

**Fig 6:**
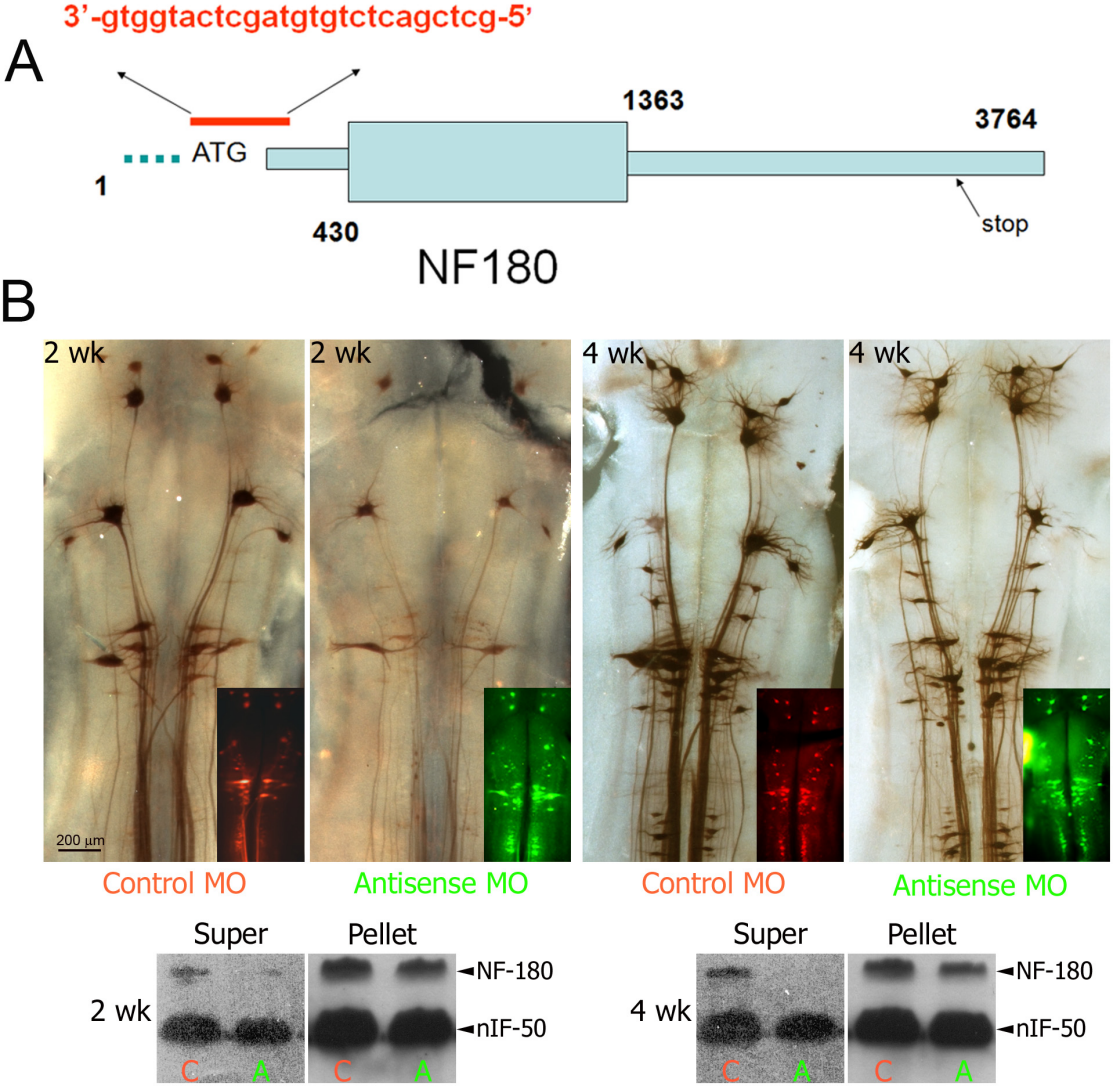
Antisense morpholino oligonucleotides (MOs) block NF180 expression in RNs. **A,** Design of the MO, the antisense sequence (in red) was complementary to the region surrounding the start codon (-4 to +21) of the NF180 gene. **B,** Fluorescently-tagged MOs were delivered to spinal-projecting neurons by retrograde transport from a spinal cord TX. After 2 or 4 wks, the brains were processed for immuno-histochemistry with LCM16, a mAb specific for NF180. Intensity of labeling was reduced in antisense MO-treated brains compared to brains treated with control MOs. Insets are fluorescent images of the same brains showing the presence of MOs. **C,** Lampreys were treated as in **B**, and the proteins extracted from the supernatant (Super) and pellet of homogenized, centrifuged brain and spinal cord rostral to the transection. Antisense MOs (A) significantly reduced the expression of NF180 in Super and pellet fractions of both 2- and 4-week groups compared with control (C). There was less effect on the pellet and no apparent effect on another neuronal intermediate filament, nIF50.

To investigate the effect of NF180 synthesis on axon regeneration, NF180 protein synthesis was inhibited by retrograde delivery of fluorescein-conjugated antisense MO complementary to the region surrounding the start codon (-4 to +21, Fig 6A). Expression of NF180 protein was monitored by whole-mount immunohistochemistry. From 2 weeks until at least 4 weeks post-TX the intensity of staining was reduced in the group treated with antisense MO compared to the group treated with control MO (Fig 6B). Western blotting of centrifuged brain homogenate fractions showed that antisense MOs reduced NF180 protein contents (Fig 6C). The reduction was more dramatic in the supernatant fraction than in the pellet fraction at both 2 and 4 weeks (Fig 6C). This result is consistent with the hypothesis that the antisense MO greatly inhibited synthesis of NF180 protein, which turns over rapidly, whereas NF180 that already had been incorporated into NFs was more stable and its concentration in the cytoplasm less affected by reduced subunit synthesis. There was no apparent effect on nIF50, another neuronal intermediate filament subunit (Fig 6C), which supports the specificity of the antisense MO.

**Fig 7:**
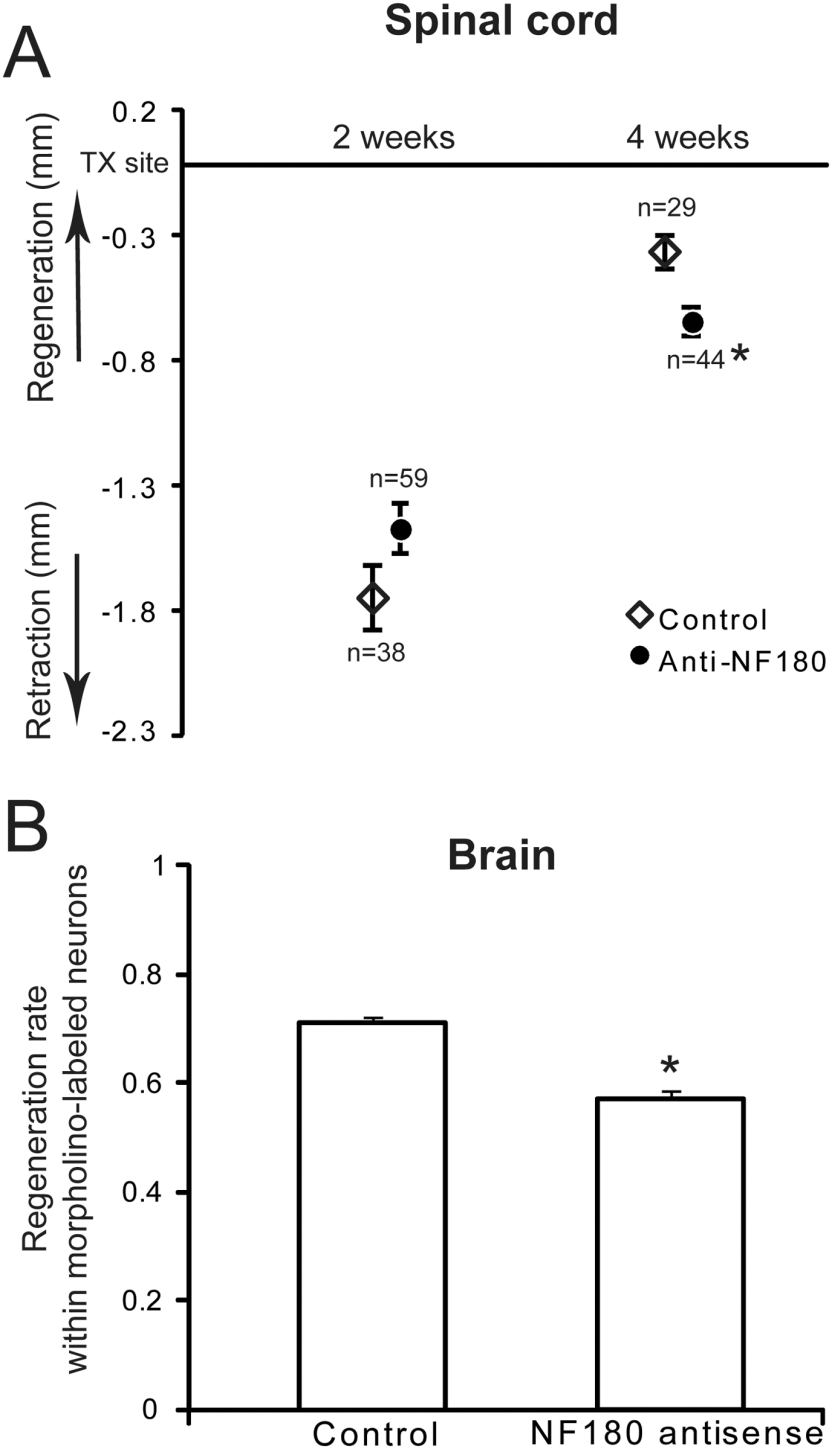
Axonal regeneration was inhibited by morpholino antisense oligonucleotides for NF180. **A,** Spinal-projecting axons were retrogradely labeled with a DTMR-conjugated control MO or a fluorescein-conjugated NF180 antisense MO. At 2 weeks post-TX, axon tips had retracted slightly farther in the control group than in the antisense group (not statistically significant). At 4 weeks, spinal-projecting axons in the control group had regenerated significantly closer to the transection site than had the antisense group (p<0.01); n = the number of axons measured. The number of animals used for each experiment is given in Table 1. Because optical isolation of individual axon tips can be difficult in these whole, living spinal cords, each animal provided only a few axons for which tip positions could be measured. Thus within treatment groups, it was more meaningful to pool axons from multiple animals in order to calculate mean axon regeneration rates, than to calculate mean regeneration rates for each animal and then compare those means. **B,** Decrease in the fraction of MO-labeled neurons whose axon had regenerated beyond the site of a second retrograde label applied 5 mm caudal to original TX at 9 weeks post-TX, and were therefore double-labeled. * indicates difference from control is significant at p<0.001 (student’s *t* test).

We evaluated the effect of NF180 synthesis on axonal retraction and early regeneration in the proximal stump by measuring the distance between projecting axon tips and the TX site at 2 and 4 weeks and comparing means of the distance in animals treated with antisense MO or control MO. The distance of axon retraction at 2 weeks post-TX was 1.59 mm ± 0.12 (mean ± SE) in controls and 1.34 mm ± 0.10 in the antisense group (P<0.10). At 4 weeks post-TX, most axons had regenerated back to the TX site in the control group, but not in the experimental group. The mean distances of axon tips from the TX site were 0.32 mm ± 0.06 in the control group and 0.57 mm ± 0.06 in the antisense group. This difference was statistically significant (P < 0.01). These results allow calculation of average regeneration rates in the proximal (rostral) stump of 91.1 μm / day for the control group and 54.5 μm / day for the antisense group. The results also suggest that inhibiting NF180 synthesis slows regeneration not by reducing initial axon retraction but by slowing subsequent elongation (Fig 7A).

At 9 weeks post-TX, the percentage of spinal projecting neurons that have regenerated was determined by inserting a second fluorescent dye into a second TX site 5 mm caudal to the original lesion. Double-labeled neurons are those to which MOs were applied and whose axons regenerated at least 5 mm past the original lesion. At 10 weeks (9 weeks recovery time plus 1 additional week to allow backfilling with the second dye), neurons with a distinct margin in which MO labeling was unambiguously distinguishable from background were counted; more faintly labeled neurons were not included. The average number of MO-labeled RNs was 61.3 ± 5.5 in controls and 66.3 ± 6.8 in the antisense group. Of these MO-labeled neurons, the number whose axons had regenerated at least 5 mm past the original TX (double labeled neurons) was 47.3 ± 3.5 in controls (77.2%) and 38.0 ± 2.7 (57.2%) in the antisense group. Thus the anti-NF180 MO group showed a 25.8% reduction in the proportion of neurons whose axons had regenerated. This difference was statistically significant at *P* < 0.001 (Fig 7B) and suggests strongly that blocking the synthesis of NF180 protein inhibits axon regeneration.

## Discussion

### Recovery of lamprey from SCI is accompanied by axonal regeneration, but not collateral sprouting

The complexity of the mammalian CNS makes it difficult to distinguish regeneration from collateral sprouting, especially in the partial lesion model of SCI. This distinction is not trivial because the two processes could be mediated by different mechanisms. For example, treatments with antibodies to Nogo and genetic knockdown of Nogo receptors and their agonists have yielded inconsistent results, and many investigators now believe that these manipulations enhance collateral sprouting, but not regeneration, of corticospinal tract (CST) axons [18] (reviewed in [19]). It is now believed that the CST is particularly refractory to all treatments tried thus far to induce regeneration, and that the appearance of new CST neurites distal to a lesion is due either to extracellular spread of tracer at the site of injection or to inability to distinguish regeneration from collateral sprouting of spared axons [20–23]. Thus many recent authors have preferred to use the term “plasticity” rather than “regeneration,” in order to indicate this uncertainty. Although collateral sprouting may contribute to functional recovery after SCI, it cannot completely replace the functionality of the lesioned axons. This is especially true in anatomically complete SCI, in which collateral sprouting cannot increase information transfer across the lesion because no spared axons span the injury.

The lamprey has served as a useful model in which to study axonal regeneration because the heterogeneous ability of its axons to regenerate spontaneously provides the opportunity to manipulate gene expression in good and bad regenerators. However, it is unknown whether collateral sprouting occurs in the injured lamprey spinal cord, as in mammals. Distinguishing true axonal regeneration from collateral sprouting is important for understanding the effect of treatments designed to enhance axon regeneration, including gene manipulations and pharmacological agents. To determine whether collateral sprouting occurs after SCI, we performed a unilateral hemisection and studied giant reticospinal axons, which normally descend unbranched through almost the entire length of cord [24]. The results showed no collateral sprouting from contralateral spared axons over a 5-week survival period, and are consistent with the idea that even in partial injury experiments, functional recovery in lamprey is based on true regeneration of severed axons.

### Role of NF180 in the structure of lamprey NFs

In mammals, NFs are obligate heteropolymers of three subunits, light (NFL), mid-sized (NFM) and heavy (NFH), in which NFL is the essential subunit, requiring either NFM or NFH to form filaments in cells [25]. It was long believed that lampreys had only one NF subunit (an NFM-like protein that we called NF180), and that therefore NFs in this species must be homopolymers [26–28]. Subsequently, it was shown that NF180 cannot self-assemble [29], and additional neuronal intermediate filament proteins were discovered, including an uncloned 50 kD protein [30], a lamprey NFL (L-NFL) and two other NFM-like proteins [31]. And as in other species, NFL was required in combination with one of the NFM-like subunits in order to form filaments [32]. The stoichiometric factors involved in assembly of lamprey NFs *in vivo* are still incompletely understood, but NF180 appears to be the most abundant subunit and forms bundles of long filaments when combined with L-NFL *in vitro* [32]. For this reason, and because we had the longest experience with it, we used NF180 as our initial target in designing MOs. However, the final MO structure targeted a region of complete identity between all known lamprey NFMs (see below) and thus should have profound effects on the ability of neurons to assemble NFs.

### Role of NF in axon regeneration

NFs traditionally have been thought to function primarily to provide structural support for the axon and to regulate axon diameter, but to play no dynamic role in axon elongation, mainly because they are scarce or absent in embryonic growth cones *in vitro*, and are expressed later in development than other neuronal cytoskeletal elements. However, several investigations suggested that NFs may be involved in elongation of axons after the initial stages of axogenesis. For example, injection of mRNA of a truncated form of NFM into frog embryos resulted in stunted axonal growth [33]. Similarly, injection of anti-NF antibodies into frog embryos stunted axon growth without preventing initial axon outgrowth [34]. Furthermore, transgenic mice lacking NFL or the combination of NFM and NFH, and thus deficient in assembled NFs, develop with a paucity of motor axons and hypoplastic peripheral nerve axons (reviewed in [35]). Thus NFs may play a more important role in axonal development than previously thought. Surprisingly, the role of NFs in regeneration has been very little studied, and in particular, little has been done to test their role in regeneration of mature CNS axons. In the present study we observed NF180 expression primarily in neurons projecting to the spinal cord. We also observed an age-dependent progressive increase in the density of NF180 immunoreactivity, and in the number of neurons expressing NF180 protein. All of these features of NF180 protein expression are consistent with previous observations on mRNA expression [17]. However, we previously demonstrated that axotomized RNs greatly reduce their expression of NF180 message in the first 4 weeks post-TX. Thereafter, NF180 mRNA expression returned toward normal selectively in good-regenerating neurons, although it was still less than pre-axotomy levels, while in bad regenerators, expression remained low for at least 10 weeks [2]. Because this pattern persisted even if axon regeneration was blocked mechanically, we postulated that NF expression might be an intrinsic characteristic of neurons that allow them to regenerate their axons. The net persistent reduction of NF180 mRNA expression even in good regenerators was a potential argument against this hypothesis, but we argued that because most of the axoplasm of RNs had been amputated by TX, and because NFs turn over slowly, less mRNA was needed to supply the remaining axons and to participate in their regeneration. This notion was supported by the observation that NF180 protein content in whole brain homogenates remained unchanged or even increased in the first two weeks post-TX. The current immunohistochemical observation that RNs maintained their intensity of NF180 immunostaining in the first 2 weeks post-TX are consistent with those previous western blot results. Reductions in NF180 protein expression were seen after 5 weeks post-TX, when some RNs showed swelling of their perikaryon and proximal dendrites. At very long recovery times, ranging from 6 months to 1 year, most of the neuronal swelling was gone, several identified RNs had disappeared, and many of the remaining neurons looked atrophied. This is consistent with an earlier study demonstrating TUNEL staining in some RNs and disappearance of others at 16 weeks post-TX, primarily affecting bad-regenerating RNs [16].

### Inhibition of NF synthesis inhibits regeneration of RN axons

The growth cones of developing axons consist of lamellipodia and filopodia, which adhere to extracellular substrata and contain surface receptors for guidance and adhesion molecules that generate intracellular signals regulating axon elongation and turning, and inhibition of axon growth [36]. Filopodia elongate by polymerization of actin microfilaments at their distal (+) ends. Lamellipodia contain actin, myosin and microtubules (MTs), but traditionally, not NFs. It is postulated that filopodia and lamellipodia exert tension on the axon through interactions between actin, myosin and the MTs, which pull the axon forward [37]. Whether this developmental mechanism also applies to regeneration of mature injured axons is not known, but in spinal-transected lampreys, regenerating axons do not appear to have growth cones [11]. Instead, axon tips are simple in shape, contain MTs and densely packed NFs, but very little F-actin [13]. Moreover, the packing density of NFs is increased in regenerating axons [12], and as described above, the regenerative abilities of identified RNs correlate with re-expression of the NF after axotomy [2]. Thus we have long postulated that NFs may play an active role in the mechanism of axon regeneration in the CNS. How this compares to the morphological and cytoskeletal features of axons regenerating in the CNS of mammals is not known, in part because regeneration in CNS is so meager. When regeneration (or collateral sprouting) has been induced by therapeutic manipulations, the shapes and cytoskeletal contents of axon tips have not been described systematically. Morphologic examination of the regenerating axon tip has been much easier in lamprey spinal cord than in the CNS of other vertebrates because the lamprey CNS is thin and lacks myelin, making it very convenient to study in wholemounts [38] and in the live animal [14]. Nevertheless, CNS axon growth in the absence of filopodia and lamellipodia is not unique to lamprey. During spinal cord development in frogs, there is a progressive simplification of growth cones [39] and it is not clear to what degree growth of axons late in development involves filopodial or lamellipodial extension. Other examples of growth without growth cones include interstitial budding of developing corticopontine projections [40], postnatal elongation of corticospinal axons into the rat cervical spinal cord [41], and early stages of optic nerve regeneration in fish [42] and frog [43]. In mammals, when optic nerve axons were induced to regenerate into grafts of peripheral nerve, the rates of transport of both NFM and β-tubulin were increased, while that for actin was decreased [44]. Thus NFs and/or MTs may play a larger role, and actin a smaller role, in regeneration than they do in early axon development *in vivo* or in 2-dimensional cultures.

An interesting model for axon growth without growth cones is the chick dorsal root ganglion cell *in vitro* with cytochalasin used to block polymerization of F-actin. Growth cones collapse but axons continue to grow, albeit more slowly [45], with simple-shaped tips, similar to those of regenerating lamprey spinal axons. This residual growth was dependent on microtubule polymerization, and not their transport function [46]. Although MTs are present in the growing tips of lamprey axons, NFs are by far the most prominent feature, and the dynamics of axon growth may be different from those of dorsal root ganglion axons with cytochalasin-collapsed growth cones. A role for NFs in promoting regeneration has been proposed, even in axons that have growth cones. In NB2a/d1 cells transfected with GFP-tagged NFM, a dynamic population of NFs appeared to be assembling at the base of the growth cone. This suggested that local NF assembly might stabilize the axon as it elongates [47]. NF180 overexpression by microinjection of plasmids into lamprey neurons resulted in neuron and axon swelling [48] but effects on regeneration have not been reported. The presence of more than one NF subunit in lamprey makes it much more difficult to over-express NFs, because stoichiometrically unbalanced overexpression of one subunit might induce toxicity, as has been seen in mammals [35, 49], possibly because NF transport requires assembly of at least NF hetero-oligomers [50]. Thus, at this point, the most effective test of the effects of NF on axonal regeneration would be to block NFs synthesis. The sequence targeted by the MO used in the present study is a region of complete identity between all three lamprey NFM-like proteins, NF180, NF132 and NF95, and should knock down all three subunits, although the highly specific antibody LCM-39 detects only NF180 on Western blots. Thus far we have been unable to find a lamprey NFH. Thus inhibition of all NFMs would be expected to reduce the ability of NFL to find a larger NF subunit partner in forming assembled NFs. Thus if NF assembly participates in the mechanism of axon elongation, our MO would be expected to inhibit the rate of axon elongation. Indeed, inhibiting NF180 synthesis slowed regeneration, not by reducing initial axon retraction but by slowing subsequent elongation. After spinal cord TX, axons first retract, and by 4 wks, many have grown back as far as the TX site. At this point, the large RN axons narrow, often branch, so that they are not easily followed in histological preparations. Instead, in the current study, regeneration was assessed at 9 weeks post-TX by retrograde transport of label applied to a second TX 5 mm caudal to the original one. We observed a 26% reduction in the probability of regeneration. This may differ from the effect of MO on how far the axons regenerated beyond the point of dye application. In our measurements of axon tip movement within the proximal stump, compared to the control MO, the NF180 antisense MO was associated with a 40% reduction in the average rate of tip advance between 2 and 4 weeks post-TX. This cannot be taken as a quantitative assessment of NF contribution to the mechanism of axon regeneration, since MOs provide a knockdown rather than a knockout of NF synthesis and might be only partially efficient. Even if it were possible to completely block synthesis of all NFM-like molecules, previously-assembled NFs might turn over slowly and act a source of dynamic NFs to stabilize axon elongation as discussed above. There also might be additional subunits that participate in NF assembly but have not yet been discovered or fully characterized. For example, a 50kD intermediate filament, NIF50, which is expressed only in neurons, was characterized biochemically [30], but has yet to be sequenced. The published lamprey genome [51] is incompletely annotated and does not identify several molecules that we have already cloned and recorded in Genbank, including NF180. Other intermediate filaments, such as α-internexin or peripherin, may well exist in lamprey, and might not be affected by our MO. Thus, the residual growth seen in the regenerating axons treated by MO may be due either to remaining intermediate filaments that are unaffected by the MO or to the contribution made by some additional growth mechanism, which may be neurofilament-based, microtubule-based, or both. Nevertheless, the present results support a role for NFs in the mechanism of axon regeneration.

## Conclusions

The present results suggest that in the lamprey, where axon regeneration after spinal cord TX is extensive, there is no evidence for collateral sprouting. This is in contrast to mammalian CNS, where true regeneration is very poor, but collateral sprouting is often robust. Although previous work showed a correlation between regenerative ability in a neuron and its ability to re-express NF180 mRNA post-axotomy after an initial downregulation, regenerative ability is not absolute. In the present study, if a reticulospinal neuron survived TX, it continued to contain NF180 protein, regardless of whether its axon had regenerated. The present results also indicate that antisense MOs are efficiently transported retrogradely from the site of injury in the spinal cord to the neuronal perikarya in the brainstem. There the MOs persist for at least 4 weeks, allowing identification of transfected neurons, and producing long-lasting reduction in NF180 expression. The inhibition of axon regeneration observed after knockdown of NF180 in RNs was not a consequence of increased initial axon retraction but represented slowed subsequent elongation. This is consistent with a possible role for NFs in the mechanism of axon regeneration in the CNS, where at least in lamprey, regeneration does not appear to involve growth cones.
